# FTIR differentiation based on genomic DNA for species identification of *Shigella* isolates from stool samples

**DOI:** 10.1038/s41598-022-06746-y

**Published:** 2022-02-17

**Authors:** Babak Pakbin, Leila Zolghadr, Shahnaz Rafiei, Wolfram Manuel Brück, Thomas B. Brück

**Affiliations:** 1 Medical Microbiology Research Center, Qazvin University of Medical Sciences, Qazvin, Iran; 2 Chemistry Department, Imam Khomeini International University, Qazvin, Iran; 3 Werner Siemens Chair of Synthetic Biotechnology, Department of Chemistry, Technical University of Munich (TUM), Lichtenberg Str. 4, 85748 Garching bei München, Germany; 4 Present Address: Institute for Life Technologies, University of Applied Sciences Western Switzerland Valais-Wallis, 1950 Sion 2, Switzerland

**Keywords:** Chemical biology, Microbiology, Molecular biology, Gastroenterology

## Abstract

Shigellosis is one of the major public health concerns in developing and low-income countries caused by four species of *Shigella*. There is an apparent need to develop rapid, cost-effective, sensitive and specific methods for differentiation of *Shigella* species to be used in outbreaks and health surveillance systems. We developed a sensitive and specific Fourier-transform infrared spectroscopy (FTIR) based method followed by principal component analysis (PCA) and hierarchical clustering analysis (HCA) assays to differentiate four species of *Shigella* isolates from stool samples. The FTIR based method was evaluated by differentiation of 91 *Shigella* species from each other in clinical samples using both gold standards (culture-based and agglutination methods) and developed FTIR assay; eventually, the sensitivity and specificity of the developed method were calculated. In summary, four distinct FTIR spectra associated with four species of *Shigella* were obtained with wide variations in three definite regions, including 1800–1550 cm^−1^, 1550–1100 cm^−1^, and 1100–800 cm^−1^ distinguish these species from each other. In this study, we found the FTIR method followed by PCA analysis with specificity, sensitivity, differentiation error and correct differentiation rate values of 100, 100, 0 and 100%, respectively, for identification and differentiation of all species of the *Shigella* in stool samples.

## Introduction

Shigellosis is a gastrointestinal infectious disease in human-caused by the invasion of the colon, rectum and ileum caused by different species of *Shigella* strains. This disease is regarded as one of the main health problems worldwide, especially in children in low-income and developing countries, due to the unhygienic conditions of water supplies and improper sanitary systems^1^. The only host for *Shigella* is human. The three main routes of infection for *Shigella* species are person-to-person, food and water, and flies with faecal contamination^2^. During the recent decades, up to 164,000 deaths annually are caused by shigellosis outbreaks worldwide, more than 55,000 of which are among the children up to 5 years of age^3^. About 60 million cases out of 188 million cases of shigellosis were children under 5 years old^1,3^. Shigellosis is caused by four species (serogroup) of *Shigella* including *S. dysenteriae* (serogroup A), *S. flexneri* (serogroup B), *S. boydii* (serogroup C) and *S. sonnei* (serogroup D), causing fever, cramp and mild to severe diarrhoea in children and adults^4^. Identification and differentiation of four species of *Shigella* are critical in preventing and managing the outbreaks caused by this pathogen^1,4^.

Culture-based methods, biochemical and serotyping tests and conventional PCR assay are used as gold standards for detecting and identifying *Shigella* species in clinical, food and water samples. These methods are costly, time-consuming, complex, and require skilled personnel to be implemented to investigate microbial outbreaks^5^. *Shigella* isolates are so sensitive that they are not viable after food or clinical sampling for a long time because of undesirable environmental conditions such as temperature and pH^6^. Diagnosis of *Shigella* species remains elusive because of low infectious doses of these pathogens, competition with other commensal bacteria and unsuitable sampling^7^. Molecular techniques in clinical, microbiological and laboratory diagnosis are divided into two main categories: sequencing-based such as 16srRNA gene sequencing, next-generation sequencing (NGS), etc.) and non-sequencing based methods, such as PCR based methods, liquid chromatography-mass spectrometry (LC–MS), biosensors, Fourier transform infrared (FTIR) spectroscopy, etc^8^. Non-sequencing based molecular methods also fall into two main groups with those associated with gene amplification, including all PCR based assays and non-amplification DNA fingerprinting methods, namely FTIR and Raman spectroscopy techniques^9^. So far, limited assays have been designed and developed to identify and discriminate different species of *Shigella* isolates from each other^10^. Some molecular methods, including MALDI-TOF MS^11^, conventional multiplex PCR^5^, LC-MS^12^, immunocapture PCR^13^, and NGS^10^ techniques, have been developed and used to differentiate four species *Shigella* isolates from clinical, food and environmental samples. It has also been shown that some standard molecular techniques such as MALDI-TOF MS and 16-rRNA gene sequencing were unable to differentiate *Shigella* species from each other as well as from Enteroinvasive *E. coli* strains^14^.

FTIR spectroscopy is a relatively cost-effective, rapid, convenient, and precise analytical technique that can reflect the DNA structure and composition^15^. FTIR is a method used to obtain an infrared spectrum of emission or absorption of a liquid, solid or gas. An FTIR spectrometer collects the high-resolution spectral data simultaneously over a wide range of spectral data. This technique identifies chemical bonds in different molecules by producing an infrared absorptions spectrum. FTIR spectroscopy has been used as a powerful tool for species identification and differentiation of eukaryotic and prokaryotic cells based on genomic DNA characterization and barcoding^16^. Spectral data from FTIR are so complicated for analysis; therefore multivariate statistical and dimension reduction methods such as principal components analysis (PCA), hierarchical clustering analysis (HCA), partial least squares (PLS) and artificial neural networks (ANN) techniques have been used for interpretation of the results^17^. Several researchers used and suggested FTIR assay followed by statistical analysis (mostly PCA and HCA assays) as a rapid, simple, relatively cheap, precise, sensitive, specific and convenient method for distinguishing the species of microbial pathogens, isolated from clinical specimens based on their genomic DNA structural differences^18^. So far, this method has not been employed to differentiate *Shigella* species isolated from clinical samples. Regarding the fact that shigellosis and *Shigella* species are now responsible for more than fifty-thousand deaths annually among children around the world and it is also highly critical and needed to cost-effectively and rapidly differentiate four species of *Shigella* isolates for investigation of shigellosis outbreaks^2^, the purpose of this study was to design and develop the FTIR assay followed by statistical analysis to identify and differentiate four species of *Shigella* isolates from stool samples.

## Results

### FTIR spectral data

**Figure 1:**
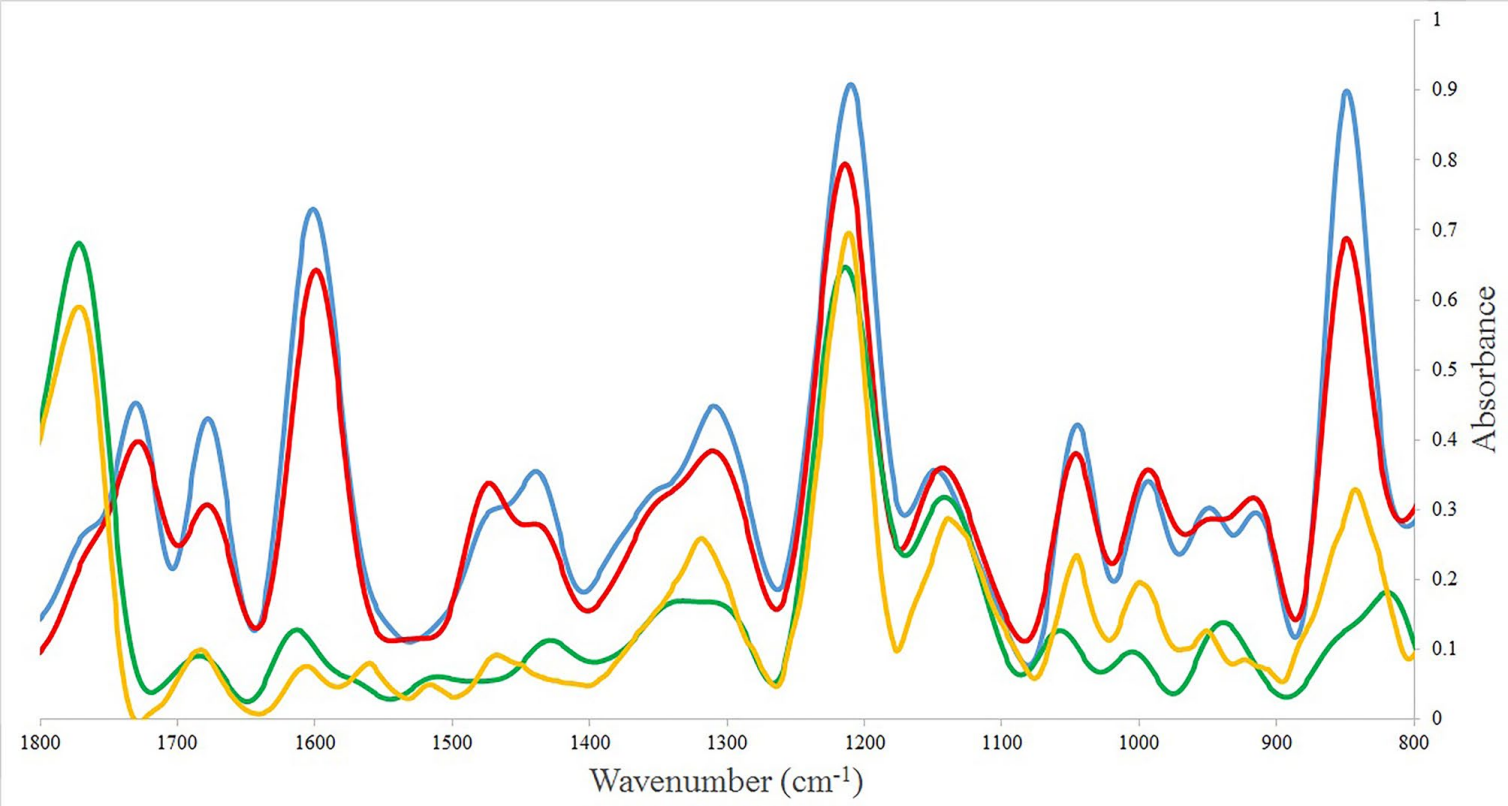
FTIR spectra of *S. dysenteriae* (red line), *S. flexneri* (blue line), *S. boydii* (green line) and *S. sonnei* (yellow line) DNA templates.

In this study, we developed an FTIR spectroscopic assay as a DNA barcoding method to differentiate four species of *Shigella* from each other. 91 *Shigella* isolates were collected from 1862 stool specimens including 18, 25, 23 and 25, *S. dysenteriae, S. flexneri, S. boydii* and *S. sonnei* isolates, respectively; using the culture and biochemical based methods and serologic tests as the gold standard methods to detect, identify and serotyping of *Shigella* species (Table 1). The FTIR spectra of the extracted DNA of the four species of *Shigella* reference strains, including *S. dysenteriae, S. flexneri, S. boydii* and *S. sonnei* were illustrated in Fig. 1. Four distinctly and significantly different FTIR spectra were observed from the DNA of the four species of *Shigella* strains to differentiate these species from each other. Comparison of the FTIR spectra of DNA from different *Shigella* species indicated significant variations in spectral characteristics in three definite regions: 1800–1550 cm^−1^, 1550–1100 cm^−1^ and 1100–800 cm^−1^. Also, prominent absorption IR marker bands were observed at 1715, 1689, 1622, 1481, 1436, 1320, 1223, 1175, 1058, 955, 866 and 845 cm^−1^. Consequently, we observed significant variations in all tested regions.

**Table 1:** *Shigella* species isolated from stool samples.

Code	Bacterial species	Strain code	Serogroup	Serotype
D1	*S. dysenteriae*	MiladH-IDD-2554	A	1
D2	*S. dysenteriae*	MiladH-IDD-2556	A	1
D3	*S. dysenteriae*	MiladH-IDD-2553	A	1
D4	*S. dysenteriae*	MiladH-IDD-2557	A	1
D5	*S. dysenteriae*	MiladH-IDD-2513	A	1
D6	*S. dysenteriae*	MiladH-IDD-2515	A	2
D7	*S. dysenteriae*	MiladH-IDD-2517	A	1
D8	*S. dysenteriae*	MiladH-IDD-2551	A	1
D9	*S. dysenteriae*	MiladH-IDD-2518	A	2
D10	*S. dysenteriae*	MiladH-IDD-2522	A	1
D11	*S. dysenteriae*	MiladH-IDD-2529	A	1
D12	*S. dysenteriae*	MiladH-IDD-2520	A	1
D13	*S. dysenteriae*	MiladH-IDD-2521	A	1
D14	*S. dysenteriae*	MiladH-IDD-2526	A	1
D15	*S. dysenteriae*	MiladH-IDD-2534	A	1
D16	*S. dysenteriae*	MiladH-IDD-2533	A	1
D17	*S. dysenteriae*	MiladH-IDD-2539	A	1
D18	*S. dysenteriae*	MiladH-IDD-2530	A	1
F1	*S. flexneri*	MiladH-IDD-2660	B	2a
F2	*S. flexneri*	MiladH-IDD-2612	B	3a
F3	*S. flexneri*	MiladH-IDD-2614	B	2b
F4	*S. flexneri*	MiladH-IDD-2610	B	2a
F5	*S. flexneri*	MiladH-IDD-2661	B	2a
F6	*S. flexneri*	MiladH-IDD-2617	B	4a
F7	*S. flexneri*	MiladH-IDD-2618	B	2a
F8	*S. flexneri*	MiladH-IDD-2651	B	4a
F9	*S. flexneri*	MiladH-IDD-2655	B	4a
F10	*S. flexneri*	MiladH-IDD-2659	B	2b
F11	*S. flexneri*	MiladH-IDD-2691	B	3a
F12	*S. flexneri*	MiladH-IDD-2602	B	2b
F13	*S. flexneri*	MiladH-IDD-2671	B	4a
F14	*S. flexneri*	MiladH-IDD-2672	B	4a
F15	*S. flexneri*	MiladH-IDD-2678	B	4a
F16	*S. flexneri*	MiladH-IDD-2693	B	4a
F17	*S. flexneri*	MiladH-IDD-2681	B	2a
F18	*S. flexneri*	MiladH-IDD-2682	B	2a
F19	*S. flexneri*	MiladH-IDD-2687	B	2b
F20	*S. flexneri*	MiladH-IDD-2684	B	4a
F21	*S. flexneri*	MiladH-IDD-2649	B	2a
F22	*S. flexneri*	MiladH-IDD-2643	B	2a
F23	*S. flexneri*	MiladH-IDD-2642	B	4a
F24	*S. flexneri*	MiladH-IDD-2640	B	4a
F25	*S. flexneri*	MiladH-IDD-2629	B	4a
B1	*S. boydii*	MiladH-IDD-2133	C	1b
B2	*S. boydii*	MiladH-IDD-2112	C	2a
B3	*S. boydii*	MiladH-IDD-2118	C	4a
B4	*S. boydii*	MiladH-IDD-2110	C	6
B5	*S. boydii*	MiladH-IDD-2114	C	6
B6	*S. boydii*	MiladH-IDD-2121	C	2b
B7	*S. boydii*	MiladH-IDD-2122	C	1b
B8	*S. boydii*	MiladH-IDD-2129	C	3a
B9	*S. boydii*	MiladH-IDD-2120	C	6
B10	*S. boydii*	MiladH-IDD-2128	C	6
B11	*S. boydii*	MiladH-IDD-2141	C	4b
B12	*S. boydii*	MiladH-IDD-2148	C	1b
B13	*S. boydii*	MiladH-IDD-2140	C	4a
B14	*S. boydii*	MiladH-IDD-2143	C	2a
B15	*S. boydii*	MiladH-IDD-2144	C	2a
B16	*S. boydii*	MiladH-IDD-2151	C	2b
B17	*S. boydii*	MiladH-IDD-2156	C	2b
B18	*S. boydii*	MiladH-IDD-2152	C	2b
B19	*S. boydii*	MiladH-IDD-2155	C	6
B20	*S. boydii*	MiladH-IDD-2164	C	2b
B21	*S. boydii*	MiladH-IDD-2165	C	2b
B22	*S. boydii*	MiladH-IDD-2169	C	4a
B23	*S. boydii*	MiladH-IDD-2171	C	3a
S1	*S. sonnei*	MiladH-IDD-2812	D	4
S2	*S. sonnei*	MiladH-IDD-2810	D	4
S3	*S. sonnei*	MiladH-IDD-2811	D	1
S4	*S. sonnei*	MiladH-IDD-2818	D	1
S5	*S. sonnei*	MiladH-IDD-2817	D	2
S6	*S. sonnei*	MiladH-IDD-2823	D	1
S7	*S. sonnei*	MiladH-IDD-2821	D	1
S8	*S. sonnei*	MiladH-IDD-2822	D	1
S9	*S. sonnei*	MiladH-IDD-2828	D	1
S10	*S. sonnei*	MiladH-IDD-2827	D	4
S11	*S. sonnei*	MiladH-IDD-2825	D	4
S12	*S. sonnei*	MiladH-IDD-2834	D	1
S13	*S. sonnei*	MiladH-IDD-2835	D	7
S14	*S. sonnei*	MiladH-IDD-2837	D	1
S15	*S. sonnei*	MiladH-IDD-2839	D	1
S16	*S. sonnei*	MiladH-IDD-2841	D	4
S17	*S. sonnei*	MiladH-IDD-2845	D	2c
S18	*S. sonnei*	MiladH-IDD-2844	D	1
S19	*S. sonnei*	MiladH-IDD-2848	D	1
S20	*S. sonnei*	MiladH-IDD-2849	D	4
S21	*S. sonnei*	MiladH-IDD-2851	D	7
S22	*S. sonnei*	MiladH-IDD-2856	D	1
S23	*S. sonnei*	MiladH-IDD-2858	D	1
S24	*S. sonnei*	MiladH-IDD-2861	D	1
S25	*S. sonnei*	MiladH-IDD-2864	D	4

### FTIR followed by PCA assay

**Figure 2:**
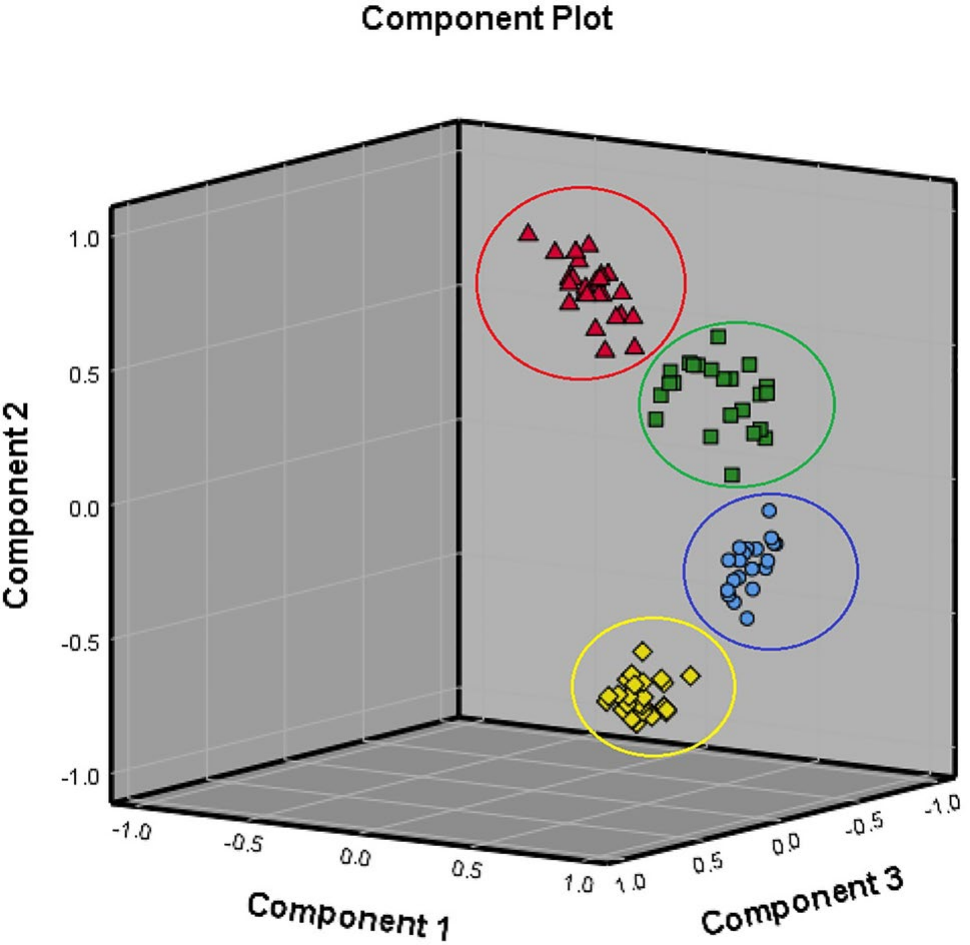
3D PCA score plot of DNA FTIR spectral data of four species of *Shigella* including *S. dysenteriae* (red tringles), *S. flexneri* (blue circles), *S. boydii* (green squares) and *S. sonnei* (yellow diamonds) isolates from stool samples.

Dimension reduction and multivariate statistical methods, including PCA and HCA assays, were used in this study to further differentiate the FTIR spectra of DNA from different species of *Shigella* reference strains and isolates collected from stool samples. Before analysis of the FTIR spectra by PCA, Bartlett's test of sphericity and the Kaiser–Meyer–Olkin (KMO) test were carried out. The results indicated that the *P*-value of Bartlett's test of sphericity was calculated at 0.000 (< 0.001), and the KMO was measured at 0.94, showing that the FTIR spectral data were significantly suitable for PCA analysis. A 3D score plot was generated using the first dominant three principal components (PCs), including PC1, PC2 and PC3, which accounted for 40.24, 34.31 and 7.18% of the total variations, respectively (Fig. 2). As it can be seen in Fig. 2, all four species of *Shigella* strains and isolates were identified and distinguished from each other successfully, and genomic DNA of different species of *Shigella* isolates was completely discriminated from each other and did not overlap in the plot. 18 out of 18, 25 out of 25, 23 out of 23 and 25 out of 25 species of *S. dysenteriae, S. flexneri, S. boydii* and *S. sonnei* isolates, respectively were correctly identified by using the FTIR method followed by PCA assay in this study showing that PCA was able to differentiate and classify the different species of *Shigella* strains. The developed method could not distinguish the serotypes of *Shigella* species from each other in this study.

### FTIR followed by HCA assay

**Figure 3:**
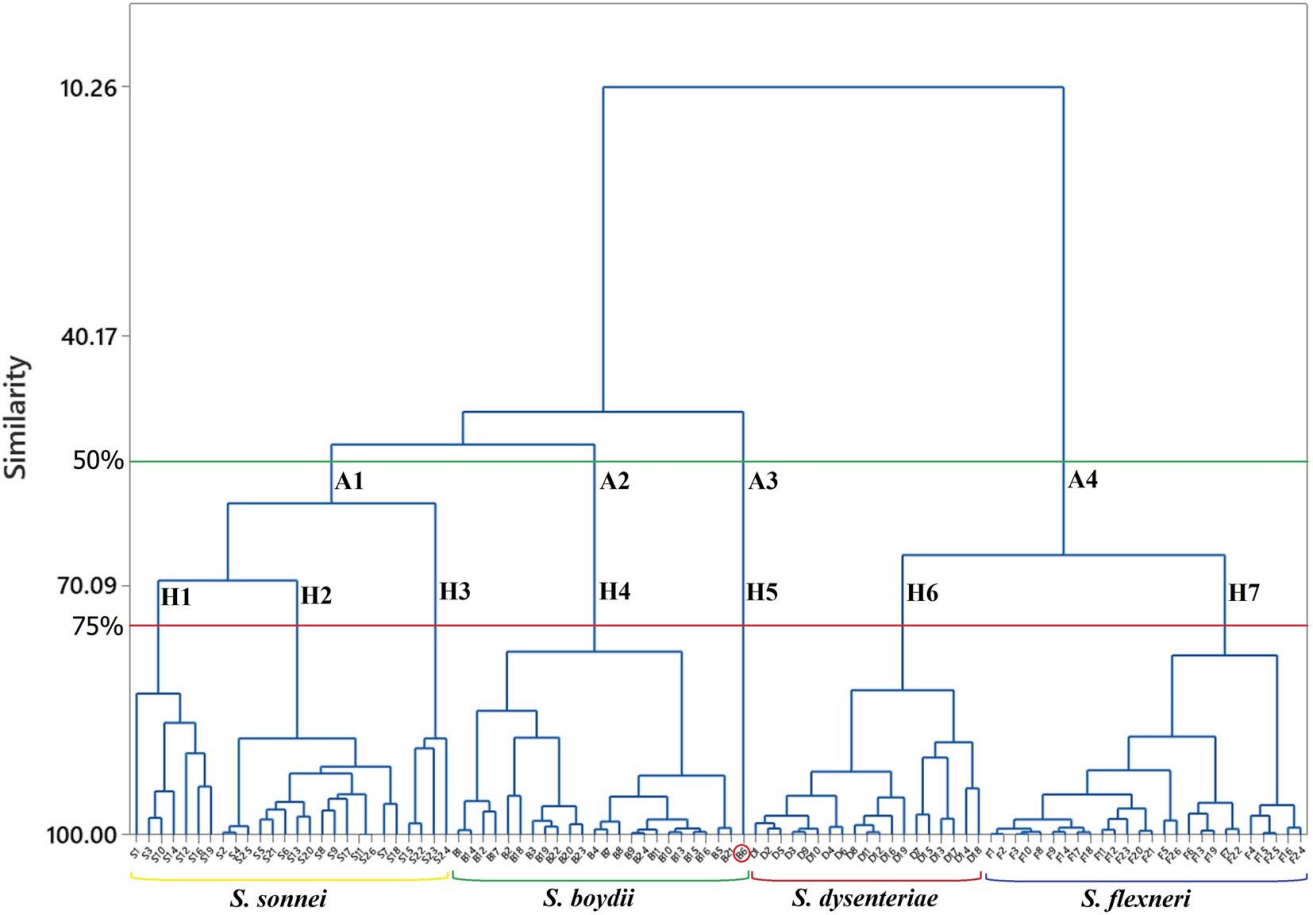
The dendrogram obtained from hierarchical clustering analysis of DNA FTIR spectral data of four species of *Shigella* including *S. dysenteriae*, *S. flexneri*, *S. boydii* and *S. sonnei* isolates from stool samples.

The Euclidean distance was used to show the linkage clustering values for hierarchical clustering and calculation of similarity measures among the FTIR spectral data of the genomic DNA of different *Shigella* species. Figure 3 showed the dendrogram form of the HCA results obtained from FTIR spectral data to identify and differentiate four species of *Shigella* isolated from stool samples in this study. We considered 50 and 75% similarity cut-offs; consequently, 4 (A1–A4) and 7 (H1–H7) major clades were recognized. In 50% similarity cut-off, *S. sonnei* and *S. boydii* isolates were differentiated into two separate clades (A1 and A2); however, *S. dysenteriae* and *S. flexneri* isolates were grouped in a single group (A4). On the other hand, in 75% similarity cut-off, *S. dysenteriae* and *S. flexneri* isolates were categorized into two distinct groups (H6 and H7), but four different clades (H1-H4) were recognized for *S. sonnei* and *S. boydii* isolates. In both 50 and 75% similarity cut-offs, one of the *S. boydii* isolates was grouped into a separate single clade (A3 or H5). As a result, whereas the HCA assay was developed to analyze the FTIR spectral data of genomic DNA of *Shigella* species using both 50 and 75% similarity cut-offs in this study, one of the *S. boydii* isolates (the isolate B6, clades A3 or H5) was identified incorrectly. At the present study, 18 out of 18, 25 out of 25, 22 out of 23 and 25 out of 25 species *of S. dysenteriae, S. flexneri, S. boydii* and *S. sonnei* isolates respectively were correctly discriminated, respectively; indicating that HCA also has the potential to be used to identify and classify the different species of *Shigella* strains. As shown in Table 2, the results of FTIR spectral data using PCA and HCA assays showed that the identification of four species of *Shigella* isolates from stool samples by FTIR method followed by PCA assay was the best, with specificity, sensitivity, differentiation error and correct differentiation rate values of 100%, 100%, 0% and 100%, respectively for all species of *Shigella* isolates. The specificity, sensitivity, differentiation error and correct differentiation rate values of FTIR analysis with HCA assay were 100%, 95.65%, 1.09% and 98.9%, respectively, for identification and differentiation of *S. boydii* isolates. However, the FTIR coupled with the HCA assay developed in this study was not capable of differentiating *Shigella* serotypes from each other.

**Table 2:** Sensitivity and specificity properties of FTIR method to differentiate four species of *Shigella* isolated from stool samples.

	*S. dysenteriae*		*S. flexneri*		*S. boydii*		*S. sonnei*	
	PCA	HCA	PCA	HCA	PCA	HCA	PCA	HCA
Specificity (%)	100	100	100	100	100	100	100	100
Sensitivity (%)	100	100	100	100	100	95.65	100	100
Differentiation error (%)	0	0	0	0	0	1.09	0	0
Correct differentiation rate (%)	100	100	100	100	100	98.9	100	100

## Discussion

*Shigella* still remains a main cause of mortality, morbidity and one of the most important communicable pathogens causing diarrhoea among infants and young children around the world^19^. Shigellosis, caused by different *Shigella* species, usually occurs as occasional outbreaks and sporadic cases in some developed and industrial countries and leads to sporadic and epidemic gastrointestinal diseases in developing and low-income countries^20^. Four species of *Shigella* cause mild to severe diarrhoea in humans. Since shigellosis is highly infectious, it is necessary to develop rapid and cost-effective methods to identify and discriminate different species of *Shigella* from each other to control and limit the outbreaks effectively^21^. Notably, classical and conventional methods for identifying pathogens in clinical and environmental samples, especially *Shigella* species, are generally time-consuming, expensive, and have low specificity and sensitivity^22^. Hence, in this study, we developed the FTIR spectroscopy method followed by PCA and HCA assays which offer sensitivity, specificity, speed and cost-effectiveness to differentiate the genomic DNA of *Shigella* species isolated from stool samples and address this problem. However, FTIR based methods require specific and expensive equipment and are relatively challenging to perform^23^ for differentiation of *Shigella* species.

The spectral data of genomic DNA of *Shigella* species were recorded in the regions between 400 and 4000 cm^−1^ and considered in the range of 1800–800 cm^−1^ (a span of 520 wavenumbers) which was previously recommended for characterization of DNA aqueous solutions for analysis by PCA and HCA assays^16^. Three distinct regions have been recognized in the FTIR spectra including 1800–1500 cm^−1^, 1500–1250 cm^−1^ and 1250–800 cm^−1^. Each section contains specific bands from sugar moiety, phosphate, base sugar, nucleobase, etc^24^. The FTIR peaks in the region of 1800–1500 cm^−1^ originating from vibrations of nucleobase and base-pairing interactions were principally assigned to the stretching vibrations of the C=O, C=N and C=C bonds in the sample^17^. The bands at 1715 cm^−1^ and 1689 cm^−1^ were assigned to the stretching vibration of guanines involved in triple helical structures and the stretch of paired guanines, respectively. The strong bands at 1622 cm^−1^ were due to C=C and C=N ring vibrations of adenine base^24^. Vibrations localized to sugar-based interactions and giving rise to the marker bands sensitive to backbone conformation, glycosidic bond rotation and sugar puckering modes were observed at the region of 1500–1250 cm^−1^^25^. The prominent absorption bands at 1481 cm^−1^, 1436 cm^−1^ and 1320 cm^−1^ were due to the adenine ring vibrations, adenine in the Z-form helices and guanine vibration in S-type sugar formation, respectively^24^. The last region between 800 cm^−1^ and 1250 cm^−1^ were due to vibrations along the sugar-phosphate chain and sensitivity to the conformation of the nucleic acid backbone. The B-form double helix of DNA appears at 1223 cm^−1^. The bands at 1175 cm^−1^ and 1058 cm^−1^ were also assigned to vibration of a sugar-phosphate backbone and backbone vibration contributing from the C–O stretch, respectively^26^. Other prominent absorption bands in the region of 1000–800 cm^−1^, including 955 cm^−1^, 866 cm^−1^ and 845 cm^−1^, were also due to the different nucleic acid N- and S-type of sugar puckering and the sugar-phosphate backbone vibrations^24^.

PCA and HCA assays were used to analyze the FTIR spectral data obtained from the genomic DNA of *Shigella* isolated from stool samples to identify and discriminate the species from each other in this study. KMO measure and the *P*-value of sphericity Bartlett`s test were observed suitable for PCA analysis^27^. PCA model for FTIR spectral data analysis indicated that all isolates species could be discriminated from each other correctly by using the PCA analysis. Dendrogram obtained from HCA analysis of the FTIR spectral data of the DNA of *Shigella* species also showed that FTIR method followed by HCA assay could be used to differentiate all species of *Shigella* from each other. However, regarding the sensitivity and specificity analysis of the assays, we found that PCA assay was more sensitive and precise for analysis of FTIR spectral data of *Shigella* isolates.

Consequently, it was demonstrated that *S. dysenteriae, S. flexneri, S. boydii* and *S. sonnei* isolated from stool samples could be well distinguished by the established FTIR-PCA protocol. FTIR method followed by multivariate statistical or dimension reduction methods has been used to discriminate the genomic DNA of the species of different eucaryotic and procaryotic cells from each other in several researches successfully. Demir et al. successfully differentiated 12 species of wild wheat from each other using attenuated total reflection FTIR followed by HCA and PCA assays. They reported both analysis methods useful and generally found FTIR method sensitive, low cost and rapid to discriminate the species of eucaryotic cells^28^. Dinkelacker et al. identified three different species of *Klebsiella* isolated from clinical samples using FTIR, MALDI-TOF and NGS methods. They showed that all of these methods could discriminate *Klebsiella* species from each other; however, FTIR showed higher discriminatory power, specificity and sensitivity to recognize three species of *Klebsiella* isolates^29^. Han et al. also used the FTIR method followed by PCA and PLS assays for species-specific analysis of the genomic DNA of meat and bone meals. They evaluated these methods to determine the source of 51 meat and bone meal samples. They developed a two-step protocol for distinguishing analysis and found the established method completely (100%) sensitive and specific^30^. Another study conducted by Potocki et al. indicated that Raman spectra-based and FTIR spectra-based molecular fingerprinting methods can be used effectively with high specificity and sensitivity to identify different species of the clinical *Candida* isolates^31^. FTIR spectroscopy is used for analytical chemistry experiments^17^. Recently, this method has been used to characterize biological substances, such as nucleic acids, to rapidly differentiate and identify different organisms in agricultural and medical sciences^32^. We have shown for the first time that FTIR-based DNA fingerprinting reflected the genomic diversity of 91 *Shigella* isolates collected from stool samples to differentiate the four species of this pathogen correctly. However, using FTIR spectroscopic method for *Shigella* species differentiation may be limited since implementing this method requires relatively expensive and specific equipment^29^. Regarding the fact that limited studies have investigated and designed practical assays for the differentiation of *Shigella* species, biosensor methods have been developed for this purpose^6,10,17,18^. Even though it was noted that these assays are expensive and complicated to implement. It is worth noting that, compared with other methods such as MALDI-TOF and agglutination assays, using FTIR and chemometrics method for bacterial identification and differentiation is highly practical and strongly useful in labs in which labs FTIR device exist and it currently is used for chemical analysis^7,12,16,27,29^. It is strongly emphasized that there is still a considerable need for developing a rapid, cost-effective, sensitive and specific methods requiring simple equipment to discriminate *Shigella* species in food, water and clinical samples.

## Conclusions

In conclusion, 91 *Shigella* strains, including 18 *S. dysenteriae*, 25 *S. flexneri*, 23 *S. boydii* and 25 *S. sonnei* were isolated from 1862 stool samples collected from patients with acute diarrhoea. FTIR method followed by PCA and HCA assays were used to analyze the DNA extracted from all *Shigella* isolates and reference strains. Four distinct and significantly different FTIR spectra reflecting four species of *Shigella* were obtained with significant variations in three definite regions, including 1800–1550 cm^−1^, 1550–1100 cm^−1^ and 1100–800 cm^−1^, to discriminate these species from each other. We found the FTIR method followed by PCA assay the best, with specificity, sensitivity, differentiation error and correct differentiation rate values of 100%, 100%, 0% and 100%, respectively, for differentiation of all species of the *Shigella* isolates from stool samples. Compared to other molecular techniques, our developed assay is more rapid, relatively cost-effective and convenient.

## Methods

### Clinical sample collection and bacterial strains

In this study, A total number of 91 *Shigella* strains including *S. dysenteriae* (n = 18), *S. flexneri* (n = 25), *S. boydii* (n = 23) and *S. sonnei* (n = 25), were used. *Shigella* strains were isolated from stool samples collected from 1862 female and male patients aged 5 to 75 years, with acute diarrhoea admitted to the Milad hospital, Tehran, Iran; September 2016 to December 2020. In this study, four species of *Shigella* were used as the reference bacterial strains and positive controls including *S. dysenteriae* ATCC 13313, *S. flexneri* PTCC 1865, *S. boydii* ATCC 12030 and *S. sonnei* PTCC 1777 were purchased from Pasteur Institute (Pasteur In., Tehran, Iran) in lyophilized form. All lyophilized reference bacterial strains were activated by inoculation in Trypticase Soy Broth (TSB, Merck, Germany) and incubation for 18 h at 37 °C. All strains were subjected to DNA extraction before the FTIR analysis.

### Isolation, identification and serotyping of *Shigella* species from stool samples

*Shigella* species were isolated from stool samples and differentiated according to the conventional methods as gold standards previously described by Mokhtari et al.^33^ and Phiri et al.^34^ Sterilized disposable inoculation loop of stool samples were directly inoculated on xylose lysine deoxycholate agar (XLD, Merck, Germany) and incubated at 37 °C for 24 h aerobically. Suspected colonies, including red ones on XLD agar morphologically resembling *Shigella* were isolated and subjected to biochemical tests. Lysine iron decarboxylase (LIA, Merck, Germany), triple sugar iron (TSI, Merck, Germany), IMViC (Indole, Methyl red, Voges-Proskauer and Citrate tests, Oxoid Ltd., UK), and urease production (Merck, Germany) tests were used to confirm the suspected colonies and identify *Shigella* in stool samples. Genus and species of each presumptive *Shigella* isolate were serologically determined and identified by slide agglutination assay using the commercial *Shigella* genus and species antisera kits (Difco Co., MI, USA), respectively. All *Shigella* isolates were serotyped by serological slide agglutination method using the *Shigella* serotyping polyvalent antisera kit (Denka Seiken, Japan) according to the manufacturers' instructions. *Escherichia coli* ATCC 25922 was used as the negative control in the isolation section. All *Shigella* isolates were stocked in TSB (Merck, Germany) containing 20% glycerol and kept at − 70 °C until further experiments.

### DNA extractions

Presumptive *Shigella* isolates on XLD agar from stool samples and the cultured reference *Shigella* strains were subjected to DNA extraction. According to the manufacturers' instructions, the genomes of the isolates and strains were extracted using the Sinaclon bacterial DNA extraction commercial kit (Sinaclon Co., Tehran, Iran). The quantity and quality of the extracted DNA were determined using the NanoDrop 2000 spectrophotometer (ThermoFisher, USA). Also, the final concentrations of all extracted DNA templates were adjusted to 20 ng µL^−1^ and they were kept at − 20 °C until further analysis.

### FTIR spectroscopy

The templates (50 µL) were first dried and then used for FTIR spectroscopy. KBr-FTIR (KBr or Potassium Bromide is used as a carrier for the sample in FTIR) technique previously described by Han et al.^30^ FTIR spectrometer (Magna 550, Madison, USA) was used over the wavenumber ranged from 4000 to 400 cm^−1^. The spectra were generated using 64 scans with a resolution of 4 cm^−1^. Analysis of each DNA template was carried out in triplicates.

### Statistical analysis

The FTIR spectral data were analyzed using dimension reduction and multivariate statistical methods by the SPSS version 26.0 software package for Windows (SPSS Inc., Illinois, USA) and the Minitab version 19 software (Minitab Inc., USA). This study used principal component analysis (PCA) and hierarchical clustering analysis (HCA) methods to analyze the FTIR spectral data obtained from the DNA templates. The Bartlett`s test of sphericity and Kaiser–Meyer–Olkin test were performed before the PCA^35^. Twenty-five types of the analysis results were selected and analyzed to evaluate the significant distribution with the infrared intensities and wavenumbers of the *Shigella* isolates spectral data. Differentiation rate of the developed method in this study was assessed according to specificity, sensitivity, differentiation error and correct differentiation rate of the assay, which were calculated as follows:

$$ {\text{Specificity}} = {\text{TN}}/\left( {{\text{TN}} + {\text{FP}}} \right) \times {1}00 $$

$$ {\text{Sensitivity}} = {\text{TP}}/\left( {{\text{TP}} + {\text{FN}}} \right) \times {1}00 $$

$$ {\text{Differentiation}}\,{\text{error}} = \left( {{\text{FP}} + {\text{FN}}} \right)/\left( {{\text{TP}} + {\text{TN}} + {\text{FP}} + {\text{FN}}} \right) \times {1}00 $$

$$ {\text{Correct}}\,{\text{differentiation}}\,{\text{rate}} = {1} - \left( {{\text{FP}} + {\text{FN}}} \right)/\left( {{\text{TP}} + {\text{TN}} + {\text{FP}} + {\text{FN}}} \right) \times {1}00 $$

where the true positive (TP) indicates the number of *Shigella* species identified by the developed method in this study and detected in the samples by the gold standard method, the false positive (FP) indicates the number of *Shigella* species which are not detected by the gold standard but identified in the samples by the developed method, the true negative (TN) indicates the number of *Shigella* species not identified and detected by the developed and gold standard methods, respectively in the samples and the false negative (FN) indicates the number of *Shigella* species which are detected by the gold standard but not identified in the samples by using the developed method in this study^30^. All measurements were carried out in triplicates.

## Ethics approval

The Ethics Committee approved the sampling and study protocols of the College of Veterinary Medicine, University of Tehran (IR.UT.REC.1394.108). At the present study, all research was performed in accordance with relevant guidelines/regulations and the Declaration of Helsinki. Also, for all cases, informed consent was obtained from the patients whose stool specimen was included in this study.

## Data Availability

All raw data included in this study are available from the corresponding author on a reasonable request.
